# Mitogen-activated protein kinases in the porcine retinal arteries and neuroretina following retinal ischemia-reperfusion

**Published:** 2010-03-10

**Authors:** Bodil Gesslein, Gisela Håkansson, Ronald Carpio, Lotta Gustafsson, Maria-Thereza Perez, Malin Malmsjö

**Affiliations:** 1 Department of Ophthalmology, Lund University, Lund, Sweden; 2 Department of Medicine, Lund University, Lund, Sweden; 3 Molecular Genetics Laboratory, Centre for Ophthalmology, Tübingen, Germany; 4 Department of Ophthalmology, University of Copenhagen, Glostrup Hospital, Glostrup, Denmark

## Abstract

**Purpose:**

The aim of the present study was to examine changes in the expression of intracellular signal-transduction pathways, specifically mitogen-activated protein kinases, following retinal ischemia-reperfusion.

**Methods:**

Retinal ischemia was induced by elevating the intraocular pressure in porcine eyes, followed by 5, 12, or 20 h of reperfusion. The results were compared to those of the sham- operated fellow eye. The retinal arteries and neuroretina were isolated separately and examined. Tissue morphology and DNA fragmentation were studied using histology. Extracellular signal-regulated kinase 1 and 2 (ERK1/2), p38, c-junNH_2_-terminal kinases (JNK), and c-jun protein and mRNA expression were examined using immunofluorescence staining, western blot, and real-time PCR techniques.

**Results:**

Pyknotic cell nuclei, terminal deoxynucleotidyl transferase dUTP nick end labeling (TUNEL)-positive cells, and glial fibrillary acidic protein mRNA expression were increased in ischemia, suggesting injury. Phosphorylated ERK1/2 protein levels were increased in the neuroretina following ischemia, while mRNA levels were unaltered. p38 protein and mRNA levels were not affected by ischemia. Immunofluorescence staining for phosphorylated p38 was especially intense in the retinal blood vessels, while only weak in the neuroretina. Phosphorylated JNK protein and mRNA were slightly decreased in ischemia. Phosphorylated c-jun protein and mRNA levels were higher in the neuroretina after ischemia-reperfusion.

**Conclusions:**

Retinal ischemia-reperfusion alters expression of mitogen-activated protein kinases, particularly ERK1/2, in the neuroretina and retinal arteries. The development of pharmacological treatment targeting these intracellular transduction pathways may prevent injury to the eye following retinal circulatory failure.

## Introduction

Retinal ischemia due to local circulatory failure in diabetes, vein thrombosis, and arterial occlusion is a major cause of sight-threatening complications and blindness [1]. Retinal ischemia leads to the formation of new blood vessels to meet the metabolic demands of the ischemic tissue. However, these newly formed blood vessels malfunction and are unable to satisfy the need for the necessary nutrients. They are not incorporated into the blood–retina barrier and will thus bleed. This causes sight-threatening complications, such as tractional retinal detachment, vitreous hemorrhage, neovascular glaucoma, and macular edema [1-3]. Today, retinal ischemia is treated with laser photocoagulation, which is effective but at the same time invasive and restores vision at the expense of large portions of the retina and its photoreceptors. Although numerous studies have been performed on ways of limiting the extent of retinal injury after ischemia, there is still no effective pharmacological treatment for this condition [2,4].

Mitogen-activated protein kinases (MAPKs) are intracellular signal-transduction pathways that have been shown to play a central role in the development of injury following ischemia in the brain and heart [5-8]. MAPK inhibitors prevent the development of pathological changes in the vasculature after both stroke and ischemic heart disease [9,10]. Ischemia induced by middle cerebral arterial occlusion in rats resulted in increased MAPK levels, which could be prevented by treatment with MAPK inhibitors [11,12]. The present study was undertaken to investigate the importance of MAPKs in the development of retinal injury following ischemia and reperfusion.

There are three main types of MAPKs: extracellular signal-regulated kinase 1 and 2 (ERK1/2), the stress-activated protein kinases (SAPKs)/c-junNH_2_-terminal kinases (JNK), and the p38 MAPKs (p38) [13,14]. The ERK1/2 pathway is primarily activated by mitogens, while the JNK/SAPK and p38 pathways are activated by stress [13,14]. MAPKs regulate gene expression, which is important in cell injury/repair and proliferation/differentiation. Studies on MAPK and retinal ischemia have recently started to appear, but these have mainly involved small animals, such as rodents, and have focused on changes in the neuroretina, not the retinal arteries [15-17]. The blood vessels of the retina are key organs in local circulatory failure, and we believe that separate analysis of the retinal vasculature is of great importance. To enable studies in human-like eyes with a vasculature that can be examined separately from the neuroretina, we have recently established a porcine model for retinal ischemia [18].

The aim of the present study was to examine changes in the MAPK intracellular signal-transduction pathways during the development of retinal injury following ischemia. Retinal ischemia was induced by elevating the intraocular pressure (IOP) in porcine eyes, followed by 5, 12, or 20 h of reperfusion. The extent of retinal injury following ischemia-reperfusion was examined by pyknotic cell nuclei counting in histological sections and terminal deoxynucleotidyl transferase dUTP nick end labeling (TUNEL) assay. Glial reactivity was assessed by quantification of glial fibrillary acidic protein (*GFAP*) mRNA levels. E*RK1/2*, *p38*, *JNK1/2/3*, and *c-jun* mRNA and protein expression were studied using immunofluorescence staining, western blot analysis, and real-time PCR techniques.

## Methods

### Ethics

All procedures and animal treatment took place in accordance with the guidelines of the Ethics Committee of Lund University, the Institute for Laboratory Animal Research (Guide for the Care and Use of Laboratory Animals), and the ARVO statement for the Use of Animals in Ophthalmic and Vision Research. The study was approved by the Lund County Administrative Court under the auspices of the Swedish Department of Agriculture.

### Animals, anesthesia, and surgical procedure

A total of 38 domestic landrace pigs of both genders, with a mean bodyweight of 70 kg each, were used for this study and were treated as described previously [18]. In brief, a 100 mg/ml intramuscular injection of ketamine (Ketaminol vet™; Farmaceutici Gellini S.p.A, Aprilia, Italy) per 15 mg/kg bodyweight, in combination with 20 mg/ml xylazine (Rompun vet™; Bayer AG, Leverkusen, Germany) per 2 mg/kg bodyweight, was used for premedication. Anesthesia was induced by continuous intravenous infusion of 20 mg/ml propofol (Diprivan™; Astra Zeneca, Södertälje, Sweden) at a dosage of 0.1–0.2 mg/kg/min in combination with intermittent fentanyl (Fentanyl B. Braun; B. Braun Melsungen AG, Melsungen, Germany) at approximately 3.5 μg/kg/h. After completion of the experiments, animals were euthanized by a lethal intravenous injection of potassium 2 mmol/kg (ADDEX Potassium Chloride, Fresenius KABI SE, Uppsala, Sweden).

Retinal ischemia was induced in one eye of each animal by raising the IOP, while the other eye served as a control. The posterior chamber of both eyes was cannulated with a 30-gauge needle. The IOP was raised to 80 mmHg in one eye by continuous infusion of a balanced salt solution for ophthalmic irrigation (AMO^™^ ENDOSOL^™^; AMO Groningen BV, Groningen, the Netherlands). The pressure was monitored using a Tono-Pen®XL tonometer (Medtronic, Jacksonville, FL). The fellow eye underwent the same surgical procedure but without elevation of the pressure. This eye is referred to as the “sham-operated eye” in the text and figures. Sixty minutes later, the cannulation needles were removed to allow reperfusion of the retinal vasculature. Ophthalmoscopic examination confirmed ischemia by the observation of the blanching of retinal arteries, which indicates that 80 mmHg is sufficient to prevent blood flow. This was confirmed directly after elevating the IOP, during ischemia (after 30 min), and at the end of the ischemic period (60 min). High IOP ischemia-reperfusion is a frequently used model for experimental retinal ischemia research [1] and has been described in several species, including rats and rabbits. High IOP produces global ocular ischemia, with obstruction of both the retinal and uveal circulation, whitening of the fundus, and iris pallor. The method is known to produce pathological features similar to those seen after central retinal artery occlusion [1]. For more details of the porcine model of experimental retinal ischemia, see Gesslein et al. [18].

### Tissue preparation

After 5, 12, or 20 h of reperfusion, both eyes of the pigs were enucleated, including the optic nerve. The eyes were dissected, the anterior segment and the vitreous humor were removed, and the eyecups were divided in half. One-half of the eyecup was used for routine histology, TUNEL assay, and immunofluorescence staining, while the retina was dissected free from adhering tissue in the other half. The large superficial retinal arteries were immediately isolated from the neuroretina by careful dissection in a balanced salt solution (AMO^TM^ ENDOSOL^TM^) for ophthalmic irrigation at 4 °C. Central and peripheral pieces of each remaining neuroretina, devoid of major vessels, were collected. The retinal and vessel samples were frozen separately at –80 °C and stored until examined using real-time PCR and western blot.

The eyes were fixed in 4% paraformaldehyde (Apoteket Farmaci AB, Hackas, Sweden) for 5 h. After fixation, the tissue was rinsed in 0.1 M Sørensen’s phosphate buffer (28 mM NaH_2_PO_4_ and 72 mM Na_2_HPO_4_; pH 7.2) and washed in the same solution with increasing concentrations of sucrose (10% to 25%). The specimens were embedded in 30% egg albumin and 3% gelatin and serially sectioned at 12 µm in a cryostat.

### Pyknotic cell count

Pyknotic cells were examined in hematoxylin and eosin-stained sections of eyes. Cells with pyknotic nuclei were counted in the inner nuclear layer (INL). For all specimens examined, the number of pyknotic cell nuclei was determined in the central and peripheral regions (0.25–2.25 mm and 9–10 mm from the optic nerve head, respectively) of the superior quadrant. The number of pyknotic cells was expressed as a cell count per 0.5 mm retina and as a percentage of the total number of cells. For each animal, two to six sections were analyzed to obtain a mean value for each animal.

### Terminal deoxynucleotidyl transferase dUTP nick end labeling staining

Sections were washed with PBS (0.14 M NaCI, 0.01 M PO_4_ Buffer, 0.003 M KCI, pH 7.45; Invitrogen, Carlsbad, CA), followed by a series of incubations with 0.05 M Tris, pH 7.6, between washing. Incubations were as follows: Tris + 0.2 µg/ml proteinase K (Invitrogen) for 5 min at 37 °C; incubation with 70% ethanol in 30% acetic acid and water (49:9:21 v/v/v) for 5 min at −20 °C; and finally a second incubation with Tris + 0.2 µg/ml proteinase K for 5 min at 37 °C. The sections were then blocked with PBS containing 10% normal goat serum, 1% bovine serum albumin (BSA), and 0.003% Triton X-100 for 1 h at room temperature. Following blocking, sections were incubated for 1 h at 37 °C with a TUNEL mixture (Cell Death Detection Kit, Roche, Penzberg, Germany) diluted 1:2 (v/v) with the blocking solution. The sections were washed with PBS and mounted with antifading mounting medium (Vectashield, Vector Laboratories Inc., Burlingame, CA).

### Immunofluorescence staining

The sections were permeabilized in a mixture of PBS and 0.25% Triton X-100 for 10 min and then blocked in PBS, 0.25% Triton X-100, 1% BSA, and 5% normal serum for 1 h at room temperature. Specimens were incubated overnight at 4 °C with PBS, 0.25% Triton X-100, 1% BSA, 2% normal serum, and the primary antibody of interest diluted (v/v): 1:200 rabbit monoclonal phosphospecific anti-ERK1/2 (#4370; Cell Signaling Technology, Inc., Danvers, MA), 1:100 rabbit polyclonal phosphospecific anti-p38 (#9211; Cell Signaling Technology), 1:200 mouse monoclonal phosphospecific anti-JNK1/2/3 (sc-6254; Santa Cruz Biotechnology, Santa Cruz, CA), 1:100 rabbit monoclonal anti-c-jun (#9165; Cell Signaling Technology), 1:100 rabbit polyclonal phosphospecific anti-c-jun (#9261; Cell Signaling Technology), 1:100 mouse monoclonal anti-protein kinase Cα (sc-8393; Santa Cruz Biotechnology), 1:200 mouse monoclonal anti-smooth muscle actin (sc-53015; Santa Cruz biotechnology), or 1:100 mouse monoclonal anti-CD31 (MCA1746; AbD Serotec, Oxford, UK). Smooth muscle actin is commonly used for the detection of smooth muscle tissue. CD31, also called platelet endothelial cell adhesion molecule 1 (PECAM-1), is expressed by various cell types but particularly by endothelial cells [19]. Sections were washed with PBS buffer and incubated with the appropriate secondary antibody (1:50 fluorescein isothiocyanate [FITC] swine antirabbit [F0205; Dako, Glostrup, Denmark] and/or 1:400 Texas Red donkey antimouse [715–076–150; Jackson ImmunoResearch, West Grove, PA]) for 1 h at room temperature. After additional washing with PBS buffer, the slides were mounted in antifading mounting medium (Vectashield; Vector Laboratories Inc.). Epitope retrieval was performed on slides stained for phosphorylated JNK by incubating in boiling hot citric acid buffer (pH 6.0) for 30 min before blocking. Sections incubated without primary or secondary antibody were used as negative controls to verify the lack of autofluorescence and unspecific secondary antibody staining.

Vertical sections including the optic nerve head were examined at the central part of the retina. The staining intensity was viewed with a light microscope equipped for fluorescence microscopy (Zeiss Axiophoto; Carl Zeiss, Oberkochen, Germany), and photographs were taken using a digital camera mounted on the microscope (Zeiss AxioCam; Carl Zeiss). For the purpose of comparisons between immunolabeled sections, sections from ischemia-reperfusion and the corresponding sham-operated eyes were processed at the same time and with the same settings to minimize variability. Antibody specificity in pig was verified either with western blot, sequence homology, and/or co-localization with more than one antibody against the same protein.

### Real-time PCR

RNA was extracted from retinal arteries and the neuroretina separately, as decribed previously [18]. Briefly, the RNA was extracted in two different ways. Samples from the sham-operated and the ischemia-reperfusion eyes of the same pig underwent the same RNA extraction procedure. Using the first technique, the tissue was homogenized with TRIzol (Invitrogen) using a metal ball and a TissueLyser (Retsch, Haan, Germany). Chloroform was added and samples centrifuged. The supernatant was transferred to new tubes and isopropanol was added to precipitate the RNA. The RNA pellet was washed with 75% ethanol, air dried and dissolved in Rnase-free water. The second technique was employed to extract RNA with an RNeasy Mini-kit (Qiagen, Valencia, CA), which allows simultaneous extraction of protein. The tissue was homogenized in RTL buffer using a metal ball and a TissueLyser. The lysate was centrifuged to remove insoluble material, and the supernatant transferred to a new tube. 70% ethanol was added, and the sample was then applied to an RNeasy minicolumn and centrifuged. The flow-through was saved for protein extraction. The column was washed with RW1 buffer and RPE buffer, and the RNA eluted with Rnase-free water. The light absorbance was measured with a spectrophotometer, and the RNA concentration and RNA/DNA ratio recorded. Reverse transcription of total RNA to cDNA was performed using the GeneAmp RNA PCR kit (Applied Biosystems, Foster City, CA) in a Perkin-Elmer DNA thermal cycler (Perkin-Elmer Applied Biosystems). First-strand cDNA was synthesized from 1 μg total RNA in a 40-μl reaction using random hexamers as primers.

Real-time PCR primers were retrieved using Primer3 software [20] or the Universal ProbeLibrary from Roche [21]. Gene-specific primer pairs were designed for each gene based on expressed sequence tags from cDNA libraries or computational gene prediction of orthologous conserved sequences (Table 1).

**Table 1:** Real-time PCR primers.

**Gene name**	**GenBank number**	**Forward primer sequence**	**Reverse primer sequence**
*GFAP*	AJ551395	CAGCGGCCCTGAGAGAGAT	TGTTAGGTCCGCAAACTTGGA
*ERK1*	AK231529	CATGACCACACTGGCTTCTT	AGCCCACAGACCAAATGTCTA
*ERK2*	NC_010456	TGACATTCAACCCTCACAAGA	ATCTGTATCCTGGCTGGAATC
*p38α*	CX057960	TGCAAGGTCTCTGGAGGAAT	CTGAACGTGGTCATCCGTAA
*JNK1*	AJ583707	TGCTTTGTGGAATCAAGCAC	TGGGCTTTAAGTCCCGATG
*JNK2*	AJ583708	TATTATCGGGCACCAGAAGTC	AACCTTTCACCAGCTCTCTCA
*JNK3*	CX061164	AGCCTGCTTCTTCTCCAGAGT	TGTGTTGGATTTGCCTTCTG
*c-jun*	DB818766	GAAAAGGAAGCTGGAGAGGAT	CTGCTGCGTTAGCATGAGTT
*ACTB*	U07786	CCTTCAACTCGATCATGAAGTGC	CGTAGAGGTCCTTCCTGATGTCC

Gene name, GenBank number and primer sequence of the primers used in the real-time PCR analyses. All primers listed are in their respective 5′- to 3′ orientation.

Real-time PCR was performed in a GeneAmp 7300 real-time PCR systems (Applied Biosystems) using the GeneAmp SYBR® Green kit (Applied Biosystems), with the cDNA synthesized above as the template in a 25 µl reaction. Real-time PCR was performed with the following profile: 1 cycle of 50 °C for 2 min, and 95 °C for 10 min followed by 40 cycles of 95 °C for 15 s, and 60 °C for 1 min. This was followed by dissociation, 1 cycle of 95 °C for 15 s, 60 °C for 30 s and 95 °C for 15 s. For more details of the reverse transcription and real-time PCR setup and running profile, see Gesslein et al. [18].

mRNA content was calculated relative to the amount of the housekeeping gene β-actin (*ACTB*). Previous analysis has shown that the use of *ACTB* and elongation factor-1α (*EF-1α*) as housekeeping genes give similar results [18]. The standard curves for each primer pair had similar slopes (3.3 for *GFAP*, 3.1 for *ERK1*, 3.3 for *ERK2*, 3.0 for *p38α*, 3.3 for *JNK1*, 3.1 for *JNK2,* 3.1 for *JNK3,* 3.6 for *c-jun,* and 3.1 for *ACTB*), indicating that the cDNA was amplified with similar efficiency.

### Western blot

Protein was extracted as previously described [18]. In brief, the flow-through was collected from RNA extraction and incubated with ice-cold acetone. The samples were centrifuged, and the supernatant was discarded. The protein pellet was air dried and resuspended in 8 M urea. The total protein concentration was determined using a Bio-Rad DC kit (Hercules, CA). Protein samples were used immediately for western blot analysis or stored at −80 °C.

Protein levels were measured in the neuroretina and in the retinal arteries. Protein samples were mixed with NuPAGE LDS sample buffer (Invitrogen, Carlsbad, CA) and incubated at 70 °C for 10 min. Equal amounts of protein (20 µg/lane) were loaded onto a NuPAGE 4%–12% Bis-Tris Gel (Invitrogen) and separated by sodium dodecyl sulfate–PAGE. A molecular weight marker (SeeBlue® Plus2; Invitrogen) was loaded onto each gel for protein band identification. After separation, the proteins were transferred to a polyvinylidene difluoride (PVDF) membrane (Invitrogen). The membrane was subsequently blocked with 5% nonfat milk in Tris-buffered saline with 0.1% Tween-20 (T-TBS) for 1 h at room temperature, and then washed with T-TBS. The membranes were incubated overnight at 4 °C with the primary antibodies of interest: 1:1,000 rabbit monoclonal phosphospecific anti-ERK1/2 (#4370; Cell Signaling Technology), 1:500 rabbit polyclonal phosphospecific anti-p38 (#9211; Cell Signaling Technology), mouse monoclonal phosphospecific anti-JNK1/2/3 (sc-6254; Santa Cruz Biotechnology), mouse monoclonal anti-ERK1/2 (#4696; Cell Signaling Technology), or 1:5,000 mouse monoclonal β-actin (sc-47778; Santa Cruz Biotechnology). Incubation was followed by washing with T-TBS. The membranes were then incubated with the appropriate secondary antibody: 1:500 swine polyclonal antirabbit Immunoglobulin G (IgG)-horseradish peroxidase (P0217; Dako) or 1:500 rabbit polyclonal antimouse IgG-horseradish peroxidase (P0260; Dako) for 2 h at room temperature, followed by washing with T-TBS. Levels of β-actin were used to confirm equal loading of the lanes. The membranes were developed using Amersham ECL Plus Western Blotting Detection Reagents (GE Healthcare, Little Chalfont, UK) and visualized using a Fujifilm LAS-1000 luminescent image analyzer (Fujifilm, Stamford, CT). Blot intensity was quantified using ImageJ software [22].

### Statistical analysis

Statistical analysis was performed using paired Student ratio *t* test with Bonferroni correction for multiple comparisons when comparing two groups and one-way ANOVA when comparing three groups or more. Calculations and statistics were performed using the GraphPad 5.0 software (GraphPad Software, Inc., La Jolla, CA). Exact p values are given in the text and figures. Values are presented as means±standard error of the mean (SEM).

## Results

### Histology and pyknotic cell nuclei count

**Figure 1:**
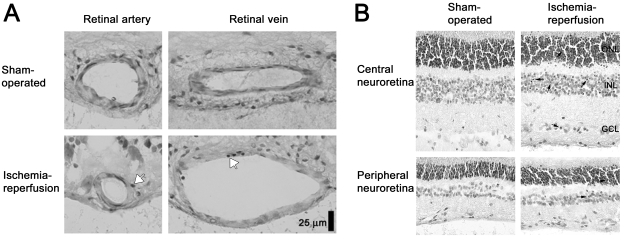
Pyknotic cells in the retinal blood vessels and neuroretina. Histopathological examination was performed by light microscopy of hematoxylin and eosin-stained sections of porcine retina from the eye exposed to ischemia-reperfusion and the sham-operated fellow eye. **A**: Artery and vein from an eye exposed to ischemia-reperfusion and the corresponding sham-operated eye. Pyknotic cell nuclei can occasionally be observed in the ischemia-reperfusion eyes (arrows) but not in the sham-operated eyes. **B**: Pyknotic cell nuclei are observed throughout the retinal sections, including the outer nuclear layer (ONL), inner nuclear layer (INL), and ganglion cell layer (GCL) of the ischemia-reperfusion eyes (arrows) but not in the corresponding sham-operated eyes.

Pyknotic cell nuclei were detected in the retinal outer nuclear layer (ONL), INL, and ganglion cell layer (GCL) from ischemia-reperfusion eyes in ten out of 17 animals (66.5±12.3 cells/0.5 mm in the INL, which equals 15.3±2.5% of the total cell count, n=10) but were virtually absent in sham-operated eyes (2.7±1.0 cells/0.5 mm in the INL, p=0.002, which equals 0.6±0.2%, p=0.0012, n=17, quantified in the INL, Figure 1). The pyknotic cell count was similar after 5 (n=5), 12 (n=7), or 20 h (n=5) of reperfusion (p=0.2616). The number of pyknotic cell nuclei was lower in the peripheral retina than in the central retina (20.0±4.2 and 66.5±12.3 cells/0.5 mm, respectively, in INL, p<0.001, which equals 4.8±1.0% and 15.3±2.5%, respectively, p=0.0012, n=10).

Pyknotic cell nuclei were detected in the vascular walls of the retinal arteries and veins from the ischemia-reperfusion eyes, while absent in the sham-operated fellow eyes (Figure 1).

### Terminal deoxynucleotidyl transferase dUTP nick end labeling staining

**Figure 2:**
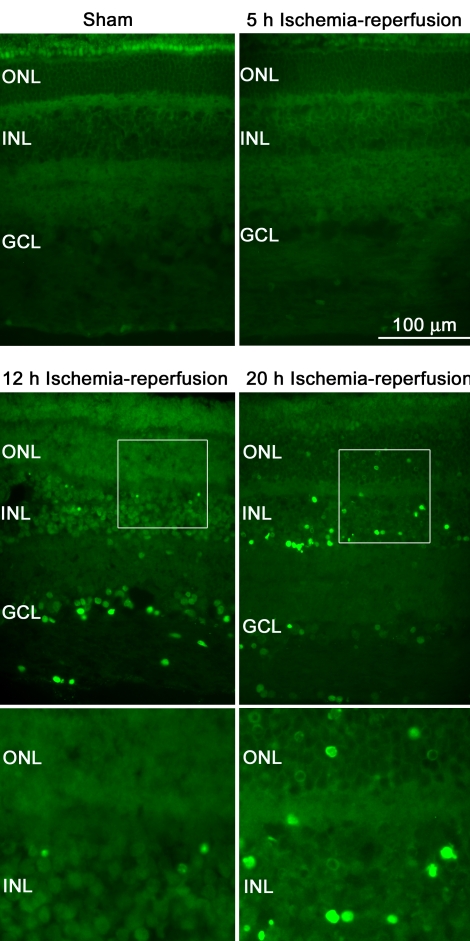
Terminal deoxynucleotidyl transferase dUTP nick end labeling (TUNEL) staining in the neuroretina. TUNEL staining of retinal sections from pigs subject to retinal ischemia followed by 5, 12, or 20 h of reperfusion versus control (sham). Note that TUNEL-positive cells can be detected in retinas from ischemia-reperfusion eyes but are absent in retinas from sham-operated eyes. The number of TUNEL-labeled cells increases gradually with the duration of reperfusion. TUNEL-positive cells are observed throughout the retinal sections, including the outer nuclear layer (ONL), inner nuclear layer (INL), and ganglion cell layer (GCL). The bottom panels are enlargements of the inserts above.

TUNEL-positive cells were observed in retinas from ischemia-reperfusion eyes, while absent in retinas from sham-operated eyes. TUNEL-positive cells were found mainly in the INL of the retina and less frequently in the GCL and ONL of the retina (Figure 2). The number of TUNEL-labeled cells increased gradually with the duration of reperfusion, being detectable after 5 h of reperfusion and more prominent after 12 and 20 h of reperfusion (Figure 2). TUNEL staining revealed cells at different stages of nuclear degeneration. At 12 h of reperfusion, most TUNEL-positive cells exhibited nuclei with round-condensed or ring-shaped staining, while at 20 h of reperfusion there were also numerous labeled fragments and cellular debris. There were less TUNEL-positive cells in the peripheral retina compared to the central retina. TUNEL-positive cells were occasionally detected in the retinal arteries and veins in the ischemia-reperfusion eyes. As for the pyknotic cell nuclei count, the number of TUNEL-positive cells varied between animals. The pigs that had high numbers of TUNEL-positive cells also had high pyknotic cell counts and vice versa.

### Glial fibrillary acidic protein mRNA expression

**Figure 3:**
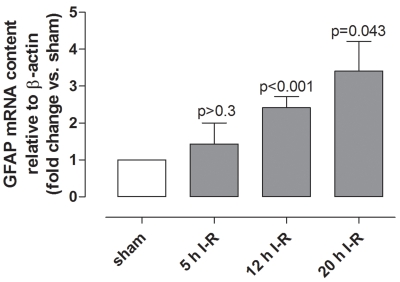
Glial fibrillary acidic protein mRNA levels in the neuroretina. Glial fibrillary acidic protein (*GFAP*) mRNA expression levels in retinas following ischemia and 5 h (n=6), 12 h (n=15), or 20 h (n=6) of reperfusion (I-R) were assessed by real-time PCR. The mRNA levels were calculated relative to the housekeeping gene β-actin (*ACTB*). The results are shown as mean values±standard error of the mean. Statistical analysis was performed using a paired Student ratio *t* test with Bonferroni correction for multiple comparisons. Note that the *GFAP* mRNA expression levels increase gradually with the duration of reperfusion.

*GFAP* mRNA expression levels were higher in the neuroretinas from ischemia-reperfusion eyes than from sham-operated eyes and increased gradually with increasing duration of reperfusion (Figure 3).

### Extracellular signal-regulated kinase 1 and 2 protein and mRNA

**Figure 4:**
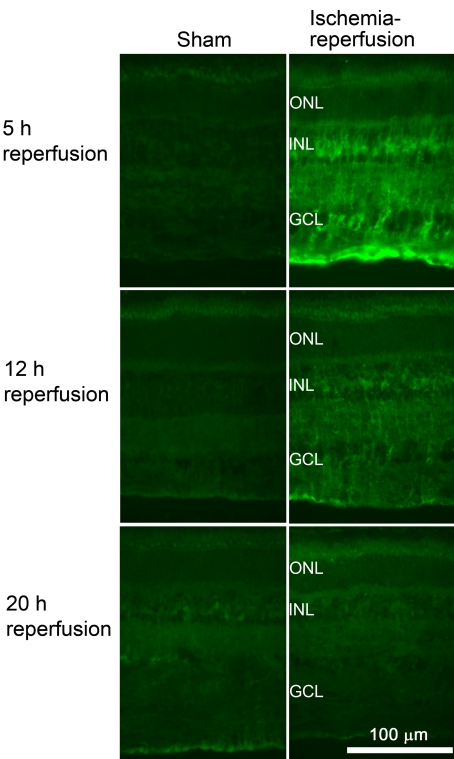
Phosphorylated extracellular signal-regulated kinase 1 and 2 (ERK1/2) immunofluorescence in the retina. Immunofluorescence staining of retina from pigs subjected to ischemia followed by 5 (n=4), 12 (n=5), or 20 (n=4) h of reperfusion and the corresponding sham-operated eyes. Note the enhanced staining for phosphorylated ERK1/2 following retinal ischemia, especially after 5 h of reperfusion. Immunofluorescence staining for ERK1/2 appears to be located in the Müller cells, including both the cell bodies and in the radial processes, and in the inner retina, probably corresponding to Müller cell endfeet and astrocytes. The abbreviations used in the figure are outer nuclear layer (ONL), inner nuclear layer (INL), and ganglion cell layer (GCL).

**Figure 5:**
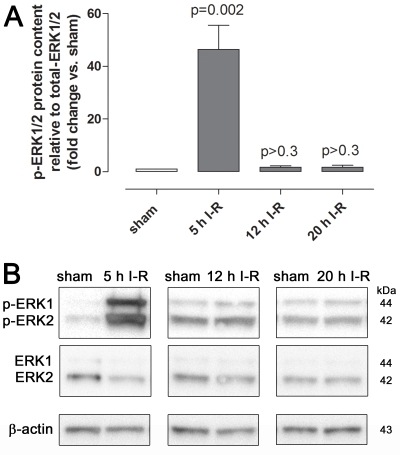
Phosphorylated extracellular signal-regulated kinase 1 and 2 (ERK1/2) protein content in the neuroretina examined using western blot. Data are presented as (**A**) mean optical density and (**B**) representative western blots from animals subjected to ischemia and 5 (n=4), 12 (n=8), or 20 (n=5) h of reperfusion (I-R) and their corresponding sham-operated eyes. Note that phosphorylated ERK1/2 (p-ERK1/2) levels were higher in the eyes subjected to ischemia followed by 5 h of reperfusion. Values are presented as means±standard error of the mean. Statistical comparison was performed using a paired Student ratio *t* test (ischemia versus sham-operated eyes) with Bonferroni correction.

**Figure 6:**
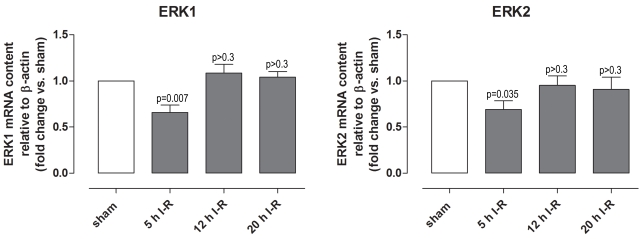
Extracellular signal-regulated kinase 1 and 2 *(ERK1 and ERK2)* mRNA levels in the neuroretina. *ERK1* and *ERK2* mRNA expression levels were assessed using real-time PCR in eyes subjected to ischemia and 5 (n=11), 12 (n=8), or 20 (n=6) h of reperfusion (I-R) in relation to sham-operated eyes. Note the initial reduction in both *ERK1* and *ERK2* after ischemia and 5 h of reperfusion. Values are presented as means±standard error of the mean. Statistical comparison was performed using a paired Student ratio *t*-test (ischemia versus sham-operated eyes) with Bonferroni correction.

Phosphorylated ERK1/2 protein expression was examined in retinal sections obtained from eyes subjected to ischemia and 5, 12, or 20 h of reperfusion and the corresponding sham-operated eyes. Phosphorylated ERK1/2 protein expression was higher in neuroretinas from eyes subjected to ischemia and 5 h of reperfusion than in sham-operated eyes, as shown by both immunofluorescence and western blot (Figure 4 and Figure 5). The difference in phosphorylated ERK1/2 staining gradually decreased over time and was slight after ischemia and 12 h of reperfusion and virtually absent after ischemia and 20 h of reperfusion (Figure 4). *ERK1* and *ERK2* mRNA levels were examined using real-time PCR. *ERK1* and *ERK2* levels were slightly reduced after ischemia and 5 h of reperfusion, whereas they remained unaltered after ischemia and 12 and 20 h of reperfusion (Figure 6).

Phosphorylated ERK1/2 staining appears to be located in the Müller cells, including both the cell bodies and the radial processes. Phosphorylated ERK1/2 staining was also observed in the inner retina, probably in the Müller cell endfeet and astrocytes. Retinal arteries did not stain for phosphorylated ERK1/2.

### p38 protein and mRNA

**Figure 7:**
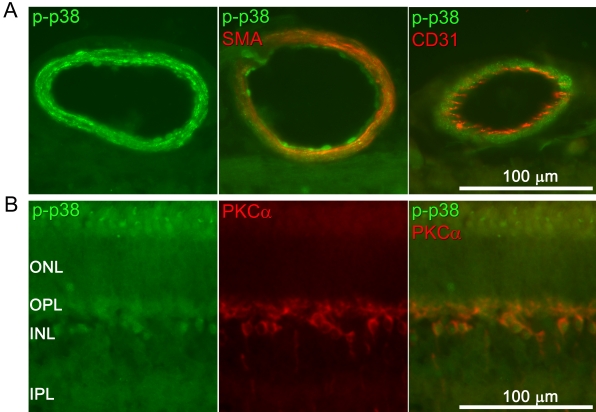
Phosphorylated p38 immunofluorescence in the retinal arteries and neuroretina. **A**: A representative example of phosphorylated p38 (p-p38; green) staining of the retinal arteries is shown. Double staining with the smooth muscle cell marker smooth muscle actin (SMA; red) and the endothelium cell marker CD31 (red) shows that p-p38 is primarily located in the smooth muscle layer. Similar results were seen for sham-operated and ischemia-reperfusion eyes. **B**: p-p38 (green) was also occasionally detected in the inner nuclear layer of the neuroretina. Double staining with protein kinase Cα (PKCα; red), a bipolar cell marker, showed that p38 was localized to bipolar cell bodies, whereas the p38 protein appears to be associated with the nucleus. The abbreviations used in the figure are outer nuclear layer (ONL), outer plexiform layer (OPL), and inner plexiform layer (IPL).

**Figure 8:**
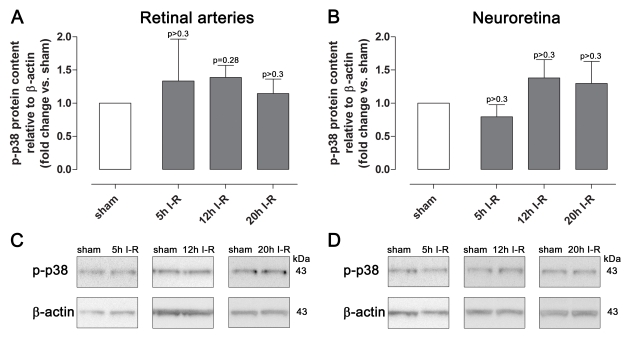
Phosphorylated p38 protein content in the retinal arteries and neuroretina. Data are presented as mean optical density (**A** and **B**) and representative western blots (**C** and **D**) from animals subjected to ischemia and 5 (n=4), 12 (n=8), or 20 (n=5) h of reperfusion (I-R) and their corresponding sham-operated eyes. Note that there was no difference in phosphorylated p38 (p-p38) expression between sham-operated and ischemia-reperfusion eyes. Values are presented as means±standard error of the mean. Statistical comparison was performed using a paired Student ratio *t* test (ischemia versus sham-operated eyes) with Bonferroni correction.

**Figure 9:**
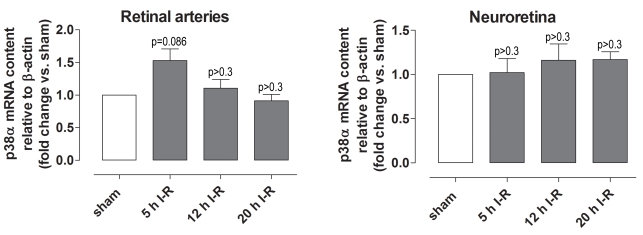
*p38α* mRNA levels in the retinal arteries and the neuroretina. *p38α* mRNA expression levels assessed with real-time PCR in eyes subjected to ischemia and 5 (n=11), 12 (n=8), or 20 (n=6) h of reperfusion (I-R) versus sham-operated eyes. Note that there was no difference in *p38α* expression between sham-operated and ischemia-reperfusion eyes. Values are presented as means±standard error of the mean. Statistical comparison was performed using a paired Student ratio *t* test (ischemia versus sham-operated eyes) with Bonferroni correction.

Immunofluorescence analysis showed staining for p38 in retinal arteries and less prominent staining in the neuroretina (Figure 7). The expression of phosphorylated p38 protein and mRNA was not affected by ischemia, according to immunofluorescence, western blot, or real-time PCR analysis (Figure 8 and Figure 9).

Double staining with an endothelial cell marker (CD31), and smooth muscle actin showed that phosphorylated p38 staining was primarily localized to the smooth muscle cell layer of the arteries (Figure 7A). Phosphorylated p38 was occasionally detected in the inner nuclear layer of the retina. Double staining with protein kinase Cα showed that phosphorylated p38 was localized to bipolar cell bodies where it appeared to stain the nucleus (Figure 7B).

### c-jun NH2-terminal kinase protein and mRNA

**Figure 10:**
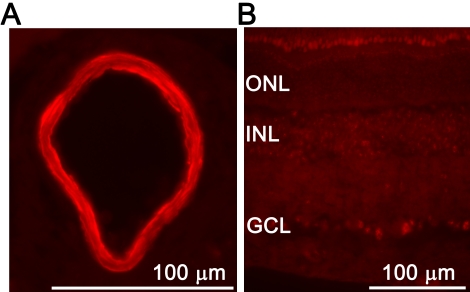
Phosphorylated c-junNH2-terminal kinase 1, 2 and 3 (JNK1/2/3) immunofluorescence in the retinal arteries and neuroretina. **A**: Representative example of phosphorylated JNK staining of the retinal arteries. **B**: Phosphorylated JNK was also detected in the ganglion cell layer (GCL) and the inner nuclear layer (INL), but not in the outer nuclear layer (ONL) of the neuroretina. Similar results were seen for sham-operated and ischemia-reperfusion eyes.

**Figure 11:**
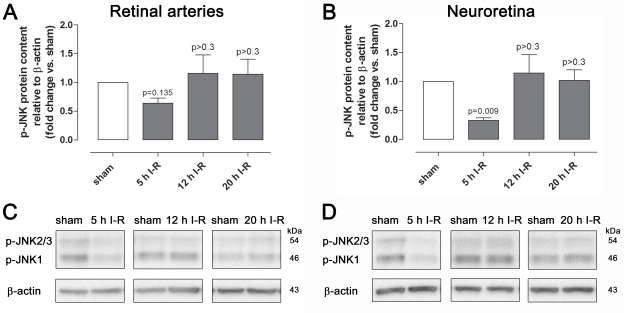
Phosphorylated c-junNH_2_-terminal kinase (JNK) protein content in the retinal arteries and neuroretina. Data are presented as mean optical density (**A** and **B**) and representative western blots (**C** and **D**) from animals subjected to ischemia and 5 (n=4), 12 (n=6), or 20 (n=5) h of reperfusion (I-R) and their corresponding sham-operated eyes. Values are presented as means±standard error of the mean. Statistical comparison was performed using a paired Student ratio *t* test (ischemia versus sham-operated eyes) with Bonferroni correction.

**Figure 12:**
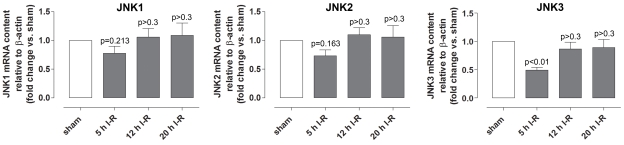
c-junNH_2_-terminal kinase 1, 2 and 3 *(JNK 1, JNK2, and JNK3)* mRNA levels in the neuroretina. *JNK1*, *JNK2,* and *JNK3* mRNA expression levels were assessed using real-time PCR in eyes subjected to ischemia and 5 (n=11), 12 (n=8), or 20 (n=6) h of reperfusion (I-R) versus sham-operated eyes. Values are presented as means±standard error of the mean. Statistical comparison was performed using a paired Student ratio *t* test (ischemia versus sham-operated eyes) with the Bonferroni correction.

Immunofluorescence analysis showed staining for p-JNK1/2/3 in retinal arteries and less prominent staining in the GCL and INL of the neuroretina (Figure 10). Quantification of phosphorylated JNK protein in retinal arteries and neuroretina with western blot showed slightly lower levels after ischemia and 5 h of reperfusion, while levels were unchanged after ischemia and 12 or 20 h of reperfusion (Figure 11). *JNK1*, *JNK2,* and *JNK3* mRNA expressions in the neuroretina were slightly decreased after ischemia and 5 h of reperfusion but unchanged after ischemia and 12 or 20 h of reperfusion (Figure 12) compared to sham-operated eyes.

### c-jun protein and mRNA

**Figure 13:**
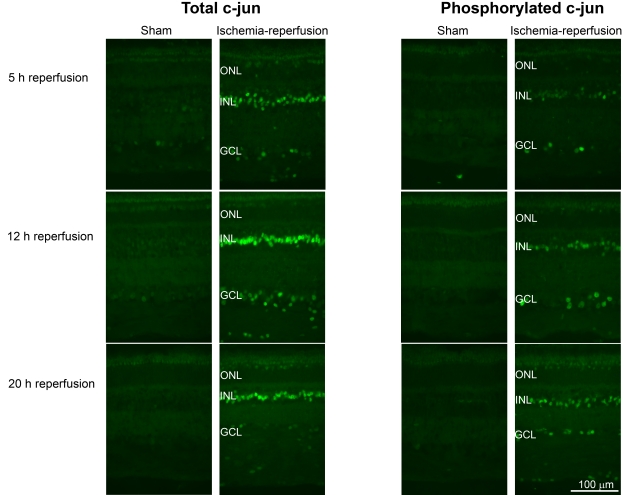
Total and phosphorylated c-jun immunofluorescence in the retina. Representative examples showing immunofluorescence staining of retina from pigs subjected to ischemia followed by 5 (n=3), 12 (n=4), or 20 (n=3) h of reperfusion and the corresponding sham-operated eyes. Note that the immunofluorescence staining intensities for both total and phosphorylated c-jun are higher in the ischemia-reperfusion eyes than in the sham-operated eyes. The abbreviations used in the figure are outer nuclear layer (ONL), inner nuclear layer (INL), and ganglion cell layer (GCL).

**Figure 14:**
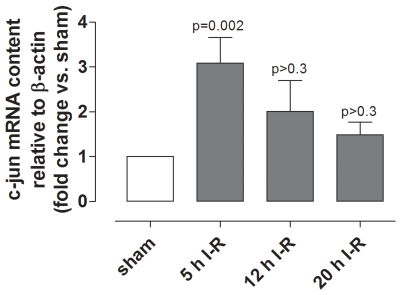
*c-jun* mRNA levels in the neuroretina. *c-jun* mRNA expression levels were assessed, using real-time PCR, in eyes subjected to ischemia and 5 (n=10), 12 (n=8), or 20 (n=6) h of reperfusion (I-R) versus sham-operated eyes. Note that the *c-jun* mRNA levels are higher in eyes subjected to ischemia and 5 h of reperfusion than in sham-operated eyes. Values are presented as means±standard error of the mean. Statistical comparison was performed using a paired Student ratio *t* test (ischemia versus sham-operated eyes) with the Bonferroni correction.

Immunofluorescence staining for total and phosphorylated c-jun was more intense in the neuroretina from eyes subjected to ischemia and 5, 12, and 20 h of reperfusion than in the neuroretina from sham-operated eyes (Figure 13). *c-jun* mRNA expression was higher after ischemia and 5 h of reperfusion and then gradually decreased over time, being less prominent after ischemia and 12 and 20 h of reperfusion (Figure 14).

Total and phosphorylated c-jun immunofluorescence staining was located in the Müller cell bodies of the INL and the ganglion cells and displaced amacrine cells of the GCL.

## Discussion

The present study was designed to examine changes in the expression of intracellular signal-transduction pathways, specifically the MAPK pathways, during the development of retinal injury following circulatory failure. A porcine model of retinal ischemia-reperfusion was used to enable detailed studies of both the retinal vasculature and the neuroretina. Experiments were performed to determine the extent of retinal damage in this model of retinal ischemia. The pig was chosen for the model since its eye has a typical primate-like architecture, including retinal blood vessels that are suitable for experimental analysis [23-25]. The results showed significant retinal ischemic injury and altered expression of MAPK. Phosphorylated ERK1/2 and c-jun levels were increased in the neuroretina following ischemia. Interestingly, phosphorylated p38 and JNK1/2/3 expression was especially high in the retinal arteries.

### Retinal cell injury and glial activation

Histopathological analysis revealed large numbers of pyknotic cell nuclei in retinas from eyes subjected to ischemia-reperfusion that were virtually absent in the sham-operated eyes. Furthermore, TUNEL-positive cells were found in the retinas from the ischemia-reperfusion eyes, indicating that irreversible damage had been produced. Evaluating the degree of retinal cell damage is a common way to quantify the degree of ischemic injury [1]. In the present study, *GFAP* mRNA levels increased in the retinas subjected to ischemia-reperfusion, indicating that an insult that triggers a molecular response had indeed taken place. It is well known that expression of intermediate filaments, such as GFAP, is increased in retinal glial cells as a reaction to injury, including ischemia [26-28]. *GFAP* levels increased early and were already present after 5 h of ischemia-reperfusion when there were only few TUNEL-positive cells. It therefore appears that *GFAP* expression is a reaction to damage of the neuronal or glial cells and not necessarily a response to neuronal cell loss. The extent of retinal injury in this model of retinal ischemia has been shown before, using a multifocal electroretinogram (mfERG) [29]. The mfERG recordings were altered following ischemia-reperfusion, with amplitudes of the mfERG waveforms being decreased and the implicit time increased [29], which is typical for retinal ischemia [30]. Taken together, the results suggest that ischemic injury had been produced, as indicated in previously established models of retinal ischemia [1].

### Extracellular signal-regulated kinase 1 and 2 in ischemia

ERK is a MAPK known to mediate cellular responses initiated by mitogens, which ultimately lead to stimulation of the transcription factors involved in differentiation and proliferation [14]. Our study and other studies have shown that the ERK pathway may also be involved in the process of cell death [6]. The results obtained in the present study show that phosphorylated ERK1/2 levels are elevated in the neuroretina following ischemia. These results are in line with previous studies in rats that showed increased phosphorylated ERK1/2 levels in the neuroretina following ischemia [15,16]. Roth et al. induced retinal ischemia in the rat by elevating the IOP and found an increase in phosphorylated ERK1/2 levels in the neuroretina. They also reported that inhibition of ERK1/2 using U0126 prevented the development of injury following retinal ischemia, as shown by a reduced number of TUNEL-positive cells, recovery of the electroretinogram b-wave, and prevention of retinal thinning 7 days after ischemia [16].

In the present study, phosphorylated ERK1/2 was detected in the Müller cells. This is in line with the results of previous studies showing phosphorylated ERK1/2 in Müller cell bodies, radial processes, and astrocytic projections following ischemia [15,16]. ERK1/2 activation has also been detected in Müller cells in several other animal models and human tissue of retinal damage, including retinal detachment [31,32] and glaucoma [33]. The present results suggest that the ERK1/2 pathway plays a role in retinal ischemia.

ERK1/2 has also been shown to play a role in the development of injury following ischemia in the brain and heart [7,8,34,35], and ERK1/2 inhibitors have been shown to prevent pathological changes in the vasculature after stroke and ischemic heart disease [9,10]. Increases in ERK1/2 phosphorylation after ischemia may be both beneficial and detrimental. ERK1/2 activity may increase inflammation by upregulating interleukin 1β, leading to necrosis, or may block apoptosis by enhancing the levels of the anti-apoptotic protein Bcl-2 [34].

### p38 in ischemia

p38 is activated by environmental stress such as oxidative stress, ischemia and inflammatory cytokines [14]. p38 has also been shown to be involved in cell death [36]. Upon activation, p38 activates transcription factors. p38 also controls the activator protein 1 (AP-1) binding site, thereby regulating the expression of genes, including c-jun [37]. In the present study, the expression levels of p38 were not affected by ischemia. Interestingly, immunofluorescence analysis showed staining for p38 in retinal arteries, while less prominent staining was seen in the neuroretina. p38 is known to mediate contractile responses to receptor agonists in vascular smooth muscle [38,39], which may explain the constitutive expression of phosphorylated p38 in the retinal arteries. Ischemia induced by middle cerebral arterial occlusion or subarachnoid hemorrhage in rats resulted in increased p38 activation in the middle cerebral artery, circle of Willis arteries, and cerebral microvessels [40,41]. In the present study no difference was seen in the levels of phosphorylated p38 in the blood vessels between sham-operated and ischemia-reperfusion eyes. The reason for this discrepancy cannot be deduced from the present study but may lie in a difference in tissue, ischemic insult, or time points studied. p38 has also been shown to be constitutively expressed in neuronal tissue. Lee et al. [42] reported constitutive expression of activated p38α and p38β in normal adult mouse brain, suggesting that the p38 pathway may not only be involved in inflammation and neuronal cell death but also in normal physiology. In the present study, p38 was localized to the bipolar cells of the INL, which is in line with previous findings showing that p38 was expressed in bipolar and amacrine cells in a rat model of retinal ischemia-reperfusion [16].

### c-junNH_2_-terminal kinase in ischemia

JNK are activated in response to a variety of noxious treatments, including heat shock, oxidant stress, reperfusion injury, and mechanical sheer stress as well as to cytokines and growth factors [43]. In the present study phosphorylated JNK1/2/3 was detected in the GCL and INL of the neuroretina. This is in line with a previous study on rat retina [16]. Phosphorylated JNK was also detected in the retinal arteries in both the sham-operated and ischemia-reperfusion eyes. Western blot experiments confirmed the presence of phosphorylated JNK in the neuroretina and retinal arteries.

### c-jun in ischemia

c-jun can form homodimers or heterodimers with c-fos to form the AP-1 transcription factor. It can also form complexes with other transcription factors, such as activating transcription factor. AP-1 transcription factor regulates the expression of genes that are important in cell injury/repair and proliferation/differentiation, such as fas ligand (FasL), tumor necros factor α (TNF-α), and cyclooxygenase-2 (COX-2) [44]. In the present study, total and phosphorylated c-jun levels were increased following ischemia-reperfusion, suggesting that c-jun plays a role in retinal ischemia. Previous studies using rats have shown similar findings. *c-jun* mRNA levels in the retina were found to be increased after ischemia induced by elevating the IOP followed by reperfusion [17]. Immunostaining for c-jun was observed in both the INL and the GCL of the neuroretina. Similarly, c-jun expression was observed by Dijk et al. in both the INL and the GCL [17].

Enhanced activity of c-jun is a common and critical event in cerebral ischemia and stroke [44]. In a mouse model of retinopathy of prematurity, c-jun was inhibited by the catalytic DNA molecule Dz13 and small interference RNA (siRNA) [45]. This inhibition reduced the neovascularization in the retina as well as decreased the vascular permeability and inflammation.

The results of the present study show that the MAPK signaling pathways ERK1/2 and c-jun were activated by retinal ischemia-reperfusion. The reason for this cannot be deduced from the present study but may depend on the many signaling pathways known to be activated by ischemia. MAPK pathways have been shown to be stimulated by several events that follow ischemia, including (1) cellular stress, such as changes in osmolarity, metabolism, and DNA damage; (2) the release of factors such as cytokines, glutamate, and growth factors; and (3) the increase in intracellular calcium and free radicals [7].

### The duration of reperfusion time

Retinal injury due to ischemia develops differently with time. In the present study, both the ERK1/2 and c-jun levels were elevated after ischemia and 5 h of reperfusion and then gradually decreased, being less prominent after ischemia followed by 12 and 20 h of reperfusion. Similar findings were observed in a study by Akiyama et al. [15] in which phosphorylated ERK1/2 levels increased early after ischemia (1 and 6 h of reperfusion) but not later (after 24 h of reperfusion). The reason for this fluctuation in ERK1/2 and c-jun levels is not known. Numerous other studies have also shown that there is an initial episode of cell death after ischemia in the retina [16,46,47] and that the degree of cell death correlates to the activity of the MAPK signaling pathways, including those of ERK1/2 and c-jun [16,48].

### Conclusions

The present animal model produced ischemic injury to the retina as verified by the presence of pyknotic cell nuclei, TUNEL-labeled cells, and increased *GFAP* mRNA levels. The MAPK signaling pathways were affected by retinal ischemia. There was an early increase in ERK1/2 and c-jun levels in the neuroretina, while JNK was slightly decreased. p38 expression was not affected by ischemia. The present results emphasize the role of MAPKs, in particular ERK1/2 and c-jun, in retinal ischemia and contribute to our knowledge concerning the intracellular signal-transduction pathways involved in the development of retinal injury following ischemia. This in turn may be useful in the identification of new targets for pharmacological treatment.
